# Anti-Glycine Receptor Antibody Mediated Progressive Encephalomyelitis with Rigidity and Myoclonus Associated with Breast Cancer

**DOI:** 10.1155/2013/589154

**Published:** 2013-07-10

**Authors:** Sofie N. De Blauwe, Patrick Santens, Ludo J. Vanopdenbosch

**Affiliations:** ^1^Department of Neurology, AZ Sint Jan Brugge Oostende, 8000 Brugge, Belgium; ^2^Department of Neurology, Universitair Ziekenhuis Gent, 9000 Gent, Belgium

## Abstract

We describe a 66-year-old woman who presented with a dramatic course of PERM. Anti-glycine receptor antibodies were found. She stabilized after plasma-exchange and partly recovered. Eighteen months later, a diagnosis of smouldering breast cancer with bone marrow metastasis was made. There are indications that this tumor was already present at first presentation. An overview of PERM and anti-glycine receptor antibodies is given.

## 1. Introduction

PERM is a severe, life-threatening condition, characterized by rigidity, painful muscle spasms, sensory and brainstem symptoms, autonomic features, breathing problems, and prominent spontaneous and stimulus-evoked myoclonus. It is part of the spectrum of stiff person syndrome and has been associated with anti-glutamic acid decarboxylase (GAD) antibodies [1]. More recently, a few cases of PERM with antibodies directed against the antiglycine receptor were reported [2–11]. 

## 2. Case Report

A 66-year-old, previously healthy retired primary school teacher, presented in October 2009 with inability to look to the left and gait instability. She also complained about dysesthesia in the left cheek with prominent painful electric tingling upon touch of the left cheek, left nostril, and left ear. This started three weeks earlier with nightly itching in the left cheek. For this reason, she had a decayed tooth removed, without resolution of the symptoms. She was not taking any medication, and her medical history was otherwise unremarkable.

Upon clinical examination, she had a horizontal gaze palsy to the left, a subtle asymmetry of expression in the left face (both eye and nasolabial fold), and had an unstable gait. There was no appendicular or truncal ataxia at that time and pyramidal signs were absent. Reflexes were normal. 

The next day, her symptoms had progressed to a one and a half syndrome and prominent appendicular ataxia. A high resolution brain MRI was strictly normal. Lumbar puncture showed 58 lymphocytes/mm^3^, 8 red blood cells/mm^3^, normal glucose of 63 mg/dL (normal 40–70 mg/dL, glycemia 91 mg/dL), normal lactate of 1.8 mmol/L (normal 1.1–2.4 mmol/L), and mildly elevated protein of 69 mg/dL (normal 20–40 mg/dL). Oligoclonal bands were negative, but there was an elevated albumine-index of 11.7 (normal < 8). IgG index was normal. 

Because of a hypothesis of rhombencephalitis, she was started on IV methylprednisone 1 g daily for three days and ceftriaxone 4 g/day, ampicillin 12 g/day, and aciclovir 1000 mg/day for 10 days. Within 3 to 4 days, she made a full recovery. 

After stopping the IV antibiotics, she was discharged. One week later though, she had a relapse. However, she presented to the ER only three weeks later. Neurological examination now demonstrated dysphagia due to a complete bulbar paralysis, a bilateral horizontal gaze palsy, hypoesthesia in the left V2-3 distribution, and loss of vibration sense in the left leg. A repeat MRI and lumbar puncture were strictly normal. The previously successful quadruple therapy was restarted upon the assumption of partially treated Listeria Monocytogenes rhombencephalitis, with the intention to treat her for 21 days. She again made a full recovery within approximately 1 week.

In the third week of the antibiotic treatment, she developed difficulty opening her mouth and stimulus-evoked trismus. When she put something in her mouth (e.g., a spoon or toothbrush), the jaw would snap shut. These spasms responded well to intravenous administration of diazepam. A few days later, she developed left hemifacial spasms and another few days later she experienced a life threatening laryngeal spasm, which was also stimulus-evoked on cough and swallow, with severe stridor, reminiscent of tetanus. She was transferred to the intensive care unit, where, two days later, she had a respiratory arrest and was successfully and quickly intubated and ventilated. Since then, she continued to have massive bilateral spontaneous and stimulus-evoked myoclonus in trunk, arms, and legs. She was conscious and apparently cognitively intact, also during bouts of severe bilateral symmetric rhythmic axial myoclonus.

Extensive blood investigation was performed. CBC was normal. ESR and C reactive protein were elevated (53 mm/h and 1,5 mg/dL, resp.). There was also a mild renal insufficiency (CrCl of 73 mg/dL, creatinine 1.2 mg/dL) and slight elevation of liver enzymes (sGPT and GGT). Thyroid function was normal and thyroid antibodies were absent. Autoimmune screening with ANA, ENA, ANCA was normal as well. Serology for Borrelia, Treponema, HIV, HSV, VZV, Mycoplasma, Toxoplasma, Bartonella, Listeria, and Clostridium tetani was negative. IgG for Chlamydia pneumoniae was strongly positive, but with negative IgM. Upon this finding, she was empirically treated with sulfamethoxazole-trimethoprim, which did not change her symptoms. 

Investigation for an occult malignancy with CT scanning of abdomen and chest was normal. Mammo-echography of the breasts was normal too, but on both chest CT and mammo-echography, there was an enlarged lymph node in the right axilla. PET scanning confirmed local elevated glucose uptake. Needle aspiration biopsy was performed with negative light microscopy. The patient stated that the lymph node had been there for over 30 years. Antineuronal antibodies (anti-Hu, anti-Yo, anti-Ri, anti-Ma, and anti-Tr), anti-GAD, anti-amphiphysin, anti-NMDA-receptor, and anti-Gq1b were negative as well. We also performed a deep duodenal biopsy for T. Whipplei, which was negative. Screening for porphyria was normal, as were urinary and serum copper levels. 

Empirical therapy with piracetam, levetiracetam, valproic acid, clonazepam, midazolam, clotiapine, gabapentin, 4-aminopyridine and several combinations of these medications was disappointing. Only carbamazepine seemed to somewhat suppress the myoclonus. Between May and July 2010, the patient underwent courses of plasma exchange, with only moderate improvement, predominantly on the myoclonus. Later, IVIg was also given, without any substantial recovery. She received a tracheostomy and percutaneous gastrostomy. After months of hospitalization first on the ICU, and later on the neurology ward, she was stable enough to be discharged to a long-term care facility. 

The sample sent for anti-NMDA-R antibodies to Professor Angela Vincent's lab in Oxford was reanalysed a couple of months after the patient was discharged and was found to be positive for anti-glycine receptor antibodies.

Eighteen months later, in December 2011, the patient was referred for removal of the percutaneous gastrostomy. She had made some spontaneous recovery. The tracheostomy had been removed earlier and the myoclonus had almost disappeared. Upon routine blood examination, a severe pancytopenia was discovered. CT of the chest and abdomen now demonstrated multiple and generalized adenopathies and suggested bone metastasis. No primary lesions could be identified. Bone marrow biopsy pointed to the diagnosis of diffuse metastasized breast cancer. CA 15.3 levels were over 1600 U/mL. Because of her general condition and the widespread disease, she was started on tamoxifen and ibandronate therapy with stable disease for about 3 months. CA 15.3 levels diminished until 1036 U/mL. The patient presented in July 2012 with dyspnea, caused by important malignant pleural effusion. CA 15.3 was up to 6400 U/mL. She was too weak for any additional treatment and eventually passed away August 2012. 

## 3. Discussion

Stiff person syndrome is a rare disorder, which presents typically with rigidity of predominantly axial and lower limb musculature and painful muscle spasms. It was first described in 1956 by Moersch and Woltman [12] as “stiff man syndrome.” As women are more frequently affected, the disorder was subsequently named stiff person syndrome (SPS). Solimena et al. demonstrated the autoimmune nature of the disorder by identifying anti-glutamic acid decarboxylase (GAD) antibodies, which are present in approximately 60% of patients with SPS [13, 14]. Often, the patient suffers from other autoimmune conditions such as type 1 diabetes, which is present in 30%–60% of patients with SPS [14, 15]. 

In some cases, SPS is paraneoplastic. In these patients, stiffness is more pronounced in neck and arms, and breast and small cell lung cancer are the most commonly encountered [16–18]. Anti-amphiphysin and anti-gephyrin antibodies have been demonstrated to accompany paraneoplastic SPS [17–19]. 

Stiff person syndrome is nowadays perceived as a spectrum of diseases, with stiff limb syndrome as a more focal and progressive encephalomyelitis with rigidity and myoclonus (PERM) as the most severe manifestation [20]. PERM consists of the same symptoms as SPS, but in addition there are sensory, brainstem, and autonomic features. Whiteley reported the first patient 20 years after Moersch and Woltman's description of SPS [21]. Cerebrospinal fluid analysis usually shows a lymphocytic pleiocytosis. Anti-GAD antibodies have been found in PERM as well [1]. More recently, a few cases of PERM were found to be associated with anti-glycine receptor antibodies [2–11].

Glycine receptors are actually chloride channels that are present mainly in the caudal pontine brainstem and spinal cord. Antibodies might disrupt glycinergic inhibition mechanisms, causing an excessive startle reflex. Though still unproven, glycine receptor antibodies might be directly pathogenic [2]. 

So far, only 13 cases of PERM with anti-glycine receptor antibodies have been described, of which 9 were men. Our patient is the fourth female patient. The youngest patient was a 14-month-old girl [10]. Two patients had thymoma, with a good response to thymectomy. No other associated malignancies were identified among the previously reported PERM patients. However, anti-glycine receptor antibodies are not exclusively linked to PERM. McKeon et al. presented 10 patients with anti-glycine receptor antibodies, with stiff person syndrome [11]. Nine of them had either SPS or stiff limb syndrome, and one had PERM. Of these, one patient with classic SPS had a Hodgkin lymphoma. Rather intriguingly, they also discovered anti-glycine receptor antibodies in a control patient with optic atrophy and inflammatory CSF. Vincent et al. also shortly reported on a patient with anti-glycine antibodies with lung cancer, although the exact phenotype of this patient is not specified [22].

Our patient was diagnosed with metastasized breast cancer 18 months after exhibiting the first symptoms and is the first patient with anti-glycine receptor antibody mediated PERM associated with breast cancer. 

Glycine receptor antibody-related PERM is part of the spectrum of neuronal surface antibody associated syndromes [23]. In contrast to the classical paraneoplastic onconeural antigens, most patients have a relatively good outcome with treatment with immunotherapies (corticosteroids, IVIg, plasma exchange, and cyclophosphamide) [24]. Our patient responded partially and temporarily to plasma exchange treatment. IVIg did not seem to make any difference. Reevaluation 18 months after discharge demonstrated that she had made a small but significant spontaneous recovery concerning the PERM-related symptoms. She eventually passed away due to the consequences of the associated malignancy. 
